# Comparing performance on the Months of the Year Backwards test in hospitalised patients with delirium, dementia, and no cognitive impairment: an exploratory study 

**DOI:** 10.1007/s41999-021-00521-4

**Published:** 2021-06-22

**Authors:** Wolfgang Hasemann, Nikki Duncan, Caoimhe Clarke, Eva Nouzova, Lisa-Marie Süßenbach, Catriona Keerie, Valentina Assi, Christopher J. Weir, Jonathan Evans, Tim Walsh, Elizabeth Wilson, Tara Quasim, Duncan Middleton, Alexander J. Weir, Jennifer H. Barnett, David J. Stott, Alasdair M. J. MacLullich, Zoë Tieges

**Affiliations:** 1 University Department of Geriatric Medicine FELIX PLATTER Basel, Burgfelderstrasse, 101 4055 Basel, Switzerland; 2 Geriatric Medicine, Edinburgh Delirium Research Group, Usher Institute, University of Edinburgh, Edinburgh, Scotland, UK; 3 Institute of Cardiovascular and Medical Sciences, University of Glasgow, Glasgow, Scotland, UK; 4 Edinburgh Clinical Trials Unit, Usher, University of Edinburgh, Edinburgh, Scotland, UK; 5 Institute of Health and Wellbeing, University of Glasgow, Glasgow, Scotland, UK; 6 Dept of Critical Care Medicine and Centre for Inflammation Research, University of Edinburgh, Edinburgh, Scotland, UK; 7 Dept of Critical Care Medicine and Anaesthesia, Royal Infirmary of Edinburgh, Edinburgh, Scotland, UK; 8 Anaesthesia, Critical Care and Pain Medicine, University of Glasgow, Glasgow, Scotland, UK; 9 Medical Devices Unit, West Glasgow Ambulatory Care Hospital, Glasgow, Scotland, UK; 10 Cambridge Cognition Ltd, Cambridge, UK; 11 SMART Technology Centre, School of Computing, Engineering and Built Environment, Glasgow Caledonian University, Glasgow, Scotland, UK

**Keywords:** Attention, Cognitive dysfunction, Delirium, Dementia, Case–control studies

## Abstract

**Purpose:**

To investigate performance of the Months of the Year Backwards (MOTYB) test in older hospitalised patients with delirium, dementia, and no cognitive impairment.

**Methods:**

Secondary analysis of data from a case–control study of 149 hospitalised patients aged ≥ 65 years with delirium [with or without dementia (*N* = 50)], dementia [without delirium (*N* = 46)], and no cognitive impairment (*N* = 53). Verbatim transcripts of MOTYB audio recordings were analysed to determine group differences in response patterns.

**Results:**

In the total sample [median age 85y (IQR 80–88), 82% female], patients with delirium were more often unable to recite months backward to November (36/50 = 72%) than patients with dementia (21/46 = 46%; *p* < 0.01) and both differed significantly from patients without cognitive impairment (2/53 = 4%; *p*’s < 0.001). 121/149 (81%) of patients were able to engage with the test. Patients with delirium were more often unable to engage with MOTYB (23/50 = 46%; e.g., due to reduced arousal) than patients with dementia (5/46 = 11%; *p* < 0.001); both groups differed significantly (*p*’s < 0.001) from patients without cognitive impairment (0/53 = 0%). There was no statistically significant difference between patients with delirium (2/27 = 7%) and patients with dementia (8/41 = 20%) in completing MOTYB to January, but performance in both groups differed (*p* < 0.001 and *p* < 0.02, respectively) from patients without cognitive impairment (35/53 = 66%).

**Conclusion:**

Delirium was associated with inability to engage with MOTYB and low rates of completion. In patients able to engage with the test, error-free completion rates were low in delirium and dementia. Recording of engagement and patterns of errors may add useful information to MOTYB scoring.

**Supplementary Information:**

The online version contains supplementary material available at 10.1007/s41999-021-00521-4.

## Key summary points

**Aim:**

To investigate performance of the Months of the Year Backwards (MOTYB) test in older hospitalised patients with delirium, dementia, and no cognitive impairment.

**Findings:**

Half of the patients with delirium (46%) could not engage with MOTYB compared to only 11% of patients with dementia without delirium. In patients able to give responses, those with delirium or dementia performed significantly worse than those without cognitive impairment.

**Message:**

Our findings show the potential value of analysing response patterns, especially initial engagement, self-correction, and ability to continue to do the task in addition to considering exclusively the capacity to correctly recite the months until July, June or January.

**Supplementary Information:**

The online version contains supplementary material available at 10.1007/s41999-021-00521-4.

## Introduction

MOTYB is a bedside test of cognitive functioning which is very widely used in clinical practice. Its popularity with clinicians stems from its speed of administration, and its accessibility and acceptability to most patients because it does not require any visual or motor capabilities except for speech and no formal testing materials are needed. MOTYB has been used to assess multiple cognitive domains, including attention and concentration [1, 2], executive function [3], working memory [4], and central processing speed [5–7].

MOTYB is a part of several delirium assessment tools [6, 8–11]. As a single item screener of delirium MOTYB shows a range of sensitivities (83–95%) and specificities (69–94%) [12, 13]. Yet there are currently limited data on MOTYB performance deficits in hospitalised patients. In particular, there are few studies that have examined the ability of MOTYB to discriminate among patients with delirium, dementia and normal cognitive functioning [14]. Such data are needed to inform clinicians as to the utility of the MOTYB in detecting delirium in research and clinical practice and to inform scoring instructions.

Current scoring methods for the MOTYB are mostly based on accuracy criteria, that is, either the number of months that patients can recite without error or using correct/incorrect performance to pre‐specified cut‐offs such as June, July or January [15, 16]. Other methods include analysis of specific response patterns, for example, omissions of two or more months, repetitions or commissions [17, 18]. The time taken to complete MOTYB has also been used as a performance measure [19]. Yet the extent to which response patterns might differ in delirium or dementia has not been reported.

In the present study, we examined MOTYB scores and response patterns in a case–control study of older hospitalised patients with delirium, dementia, and no cognitive impairment. We hypothesized that patients with delirium (with or without dementia) would show lower scores and show different response patterns than patients with dementia (without delirium) or no cognitive impairment.

## Methods

### Design, setting, and participants

This study is a secondary post hoc analysis. As such these analyses are exploratory and should be considered hypothesis-generating rather than confirming. Data were derived from a case–control study of a software application for detecting attention deficits in delirium, and the recruitment and assessment methods are described in detail in the original study [20]. Briefly, patients were recruited from geriatrics and orthopaedics wards of the Royal Infirmary of Edinburgh and Glasgow Royal Infirmary, Scotland. Potential participants were first identified through consultation with the clinical care team. Patients were included if they were aged 65 years and above.

Cases and controls were frequency-matched by age within 10-year age bands and sex. Three groups of patients were recruited: patients with delirium (with or without dementia), patients with a diagnosis of dementia (without delirium), and patients without cognitive impairment. Exclusion criteria were visual or hearing impairments severe enough to preclude testing, non-fluent English speakers and photosensitive epilepsy. The study was approved by the Scotland A Research Ethics Committee.

### Enrolment

Five researchers, all psychology graduates, carried out the recruitment and assessment of participants (ND, CC, LS, EN, and ZT). The researchers were fully trained in the use of all cognitive assessments and applying the Diagnostic and Statistical Manual of Mental Disorders-5 (DSM-5) criteria [21]. Written informed consent was obtained from patients with sufficient capacity to understand their involvement in the study. In the case of insufficient capacity, an appropriate legal proxy was contacted to provide informed consent.

### Assessments and participant groupings

The reference standard assessment for delirium based on DSM-5 criteria was informed by the Delirium Rating Scale—Revised 98 [DRS-R98 [22]]. As part of the assessment several scales and cognitive tests were administered: The Observational Scale of Level of Arousal [OSLA, [23]], the Richmond Agitation–Sedation Scale [RASS [24]], the Short Orientation, Memory and Concentration Test [OMCT [25]] and the Abbreviated Mental Test [AMT10 [26]], MOTYB, Days of the Week Backwards, Counting Backwards from 20 to 1, and Digit Span [20].

The following process for MOTYB was used. Patients were asked to recite the months of the year in backwards order starting with December. Task instructions were repeated once if the participant did not understand the task or if they started reciting the months of the year forward. No further prompts were allowed. Responses to MOTYB were recorded and transcribed (see below). Patients who were unable to engage meaningfully with MOTYB due to severe cognitive impairment and/or disturbances in arousal (as judged by the researchers) were retained for the analysis.

Patients were classified as having dementia through either a prior formal clinical diagnosis of dementia, or if they met DSM-IV criteria for dementia (using information from case notes and informants) as determined by a consultant geriatrician [27].

Patients for whom an OMCT score > 20 was obtained and who did not have an acute change from baseline or a diagnosis of dementia were grouped as having no cognitive impairment.

Using the above assessments, including MOTYB test results, participants were categorized into three groups: delirium according to DSM-5 diagnostic criteria (with or without dementia), dementia (without delirium), or no cognitive impairment. When grouping was unclear, a decision was sought in discussion with experienced geriatricians (AMJM and DS). When participants could not be classified into one of the predefined clinical groups, a patient was declared as indeterminate and excluded from the analysis.

### MOTYB transcript analysis

The five researchers who carried out the recruitment and assessment of participants (ND, CC, LS, EN, and ZT) audio-recorded the MOTYB task. To reduce bias during the transcription process recordings were transcribed blindly by a second researcher who had not carried out the reference standard assessment battery and was not aware of the categorization of the participant. Researcher ND then assessed all transcripts blind to the categorizations and recorded the type of errors made.

Based on the framework used by Meagher et al. [7], the following responses were considered errors: omissions, commissions in the wrong place, non-relevant commissions and repetitions. Following inspection of the transcripts, we added the following response patterns: self-corrections, reciting the months forward, stopping part way through the task and not being able to meaningfully engage with the task (see online Table 1 for examples).

### Statistical analysis

Descriptive and inferential statistical analyses were conducted using IBM SPSS Statistics version 22 (IBM, Inc., Chicago, IL). Fisher’s exact tests and Mann–Whitney *U* tests were used to analyse group differences. Holm–Bonferroni corrections were applied to account for multiple comparisons [28].

## Results

### Participants

**Fig. 1:**
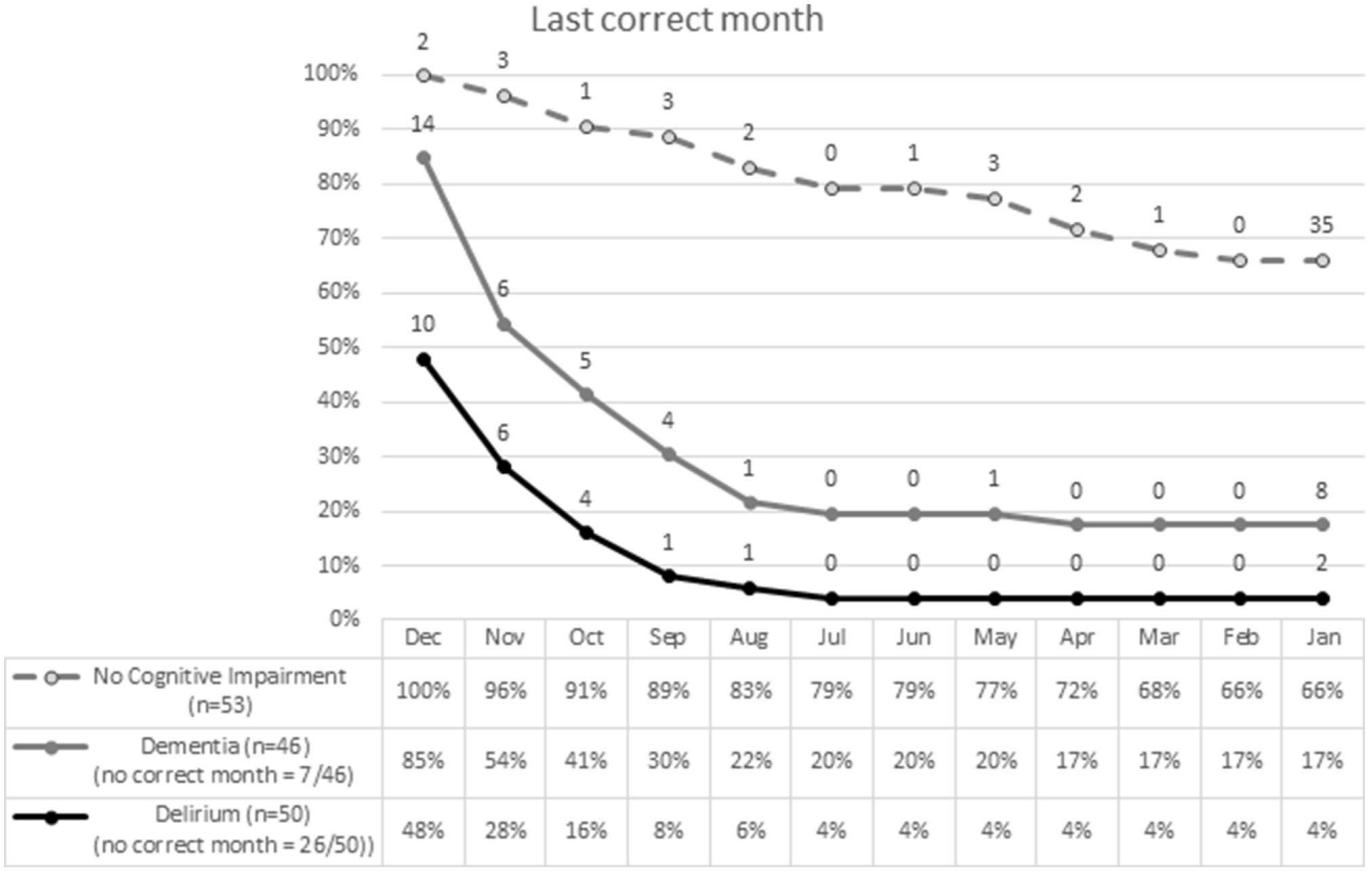
Last correct month in MOTYB in 149 patients. 2 patients without cognitive impairment, 14 with dementia and 10 with delirium (with and without dementia) stated December as the last correct month in MOTYB. Additionally, out of 50 patients with delirium, 26 could not state December as the last correct month. Of those 3 were able and 23 were unable to meaningfully engage with MOTYB. Out of 46 patients with dementia (without delirium), 7 could not state December as the last correct month. Of those, 2 were able and 5 were unable to meaningfully engage with MOTYB

From 187 hospitalised older patients, 170 MOTYB audio recordings were available. From the initial sample of 170 patients with MOTYB recordings, 21 patients were grouped as indeterminate and were excluded from further analysis. The analysis dataset for this study thus consisted of 149 patients: 50 patients with delirium, 46 patients with dementia, and 53 patients without cognitive impairment (Online-Fig. 1).

Patients were aged between 67 and 98 years (median 85 years, inter-quartile range (IQR) 80–88). Patients without cognitive impairment (median 82 years, IQR 76–85) were younger compared to patients with dementia (median 85 years, IQR 82–89; *U* = − 3.640, *p* < 0.001) and patients with delirium (with or without dementia) (median 87 years, IQR 83–90; *U* = − 4.194, *p* < 0.001). Delirium and dementia groups did not differ in age (*U* = − 0.889, *p* = 0.374). The majority of patients (122/149) were female [82% (Table 1)].

**Table 1:** Patient characteristics

	Total	Delirium (with or without pre-existing cognitive impairment)	Dementia (no delirium)	No cognitive impairment
*N*	149	50	46	53
Age median (IQR)	85.0 (80.0; 88.0)	87.0 (82.5; 90.0)**	85.0 (82,0 89.0)**	82.0 (76.0; 85.0)
Gender female (%)	122 (81.9)	39 (78.0)	35 (76.1)	48 (90.6)
CCI	3.0 (2.0; 5.0)	4.0 (2.0; 5.0)	3.0 (2.0; 5.0)	3.0 (1.0; 4.25)
Short OMCT (score) (median, IQR) Normal (*N*, %) Minimal cognitive impairment (*N*, %) Severe cognitive impairment (*N*, %)	11 (3–25) (n = 144)	3 (0–6)** (*n* = 47) 2 (4%) 7 (15%) 38 (81%)	6 (2.25–12)** (*n* = 44) 4 (9%) 15 (34%) 25 (57%)	26 (23.5–28) (*n* = 53) 52 (98%) 1 (2%)
AMT10 (score) (median, IQR)	5 (2–8) (*n* = 140)	1 (0–4)** (*n* = 45)	3 (2–6)**^†^ (*n* = 42)	9 (8–10) (*n* = 53)
Brief Attention Test (score) (median, IQR)	4 (3–6) (*n* = 141)	2.5 (0–4)** (*n* = 46)	4 (3–5)**^†^ (*n* = 44)	6 (6–7) (*n *= 51)
DRS-R98 total (score) (median, IQR)	8 (1–18) (*n* = 147)	20 (16–23)** (*n* = 49)	9 (6–12)**^†^ (*n* = 45)	1 (0–1) (*n* = 53)
DRS-R98 severity (score) (median, IQR)	7 (1–13.75) (*n* = 148)	16 (11–19) ^**^ (*n* = 50)	8 (6–11)**^†^ (*n* = 45)	1 (0–1) (*n* = 53)
OSLA (score) (median, IQR)	0 (0–2.5) (*n* = 149)	4 (2–6)** (*n* = 50)	0 (0–1)**^†^ (*n* = 46)	0 (0–0) (*n* = 53)

*IQR* Inter-quartile range, *CCI* Charlson Comorbidity Index [32]

*Short OMCT* Short Orientation-Memory-Concentration Test (score range 0–28). Short OMCT categories: severe cognitive impairment (score 0–8), minimal impairment (score 9–20), normal (score > 20)

*AMT10* Abbreviated Mental Test-10 (score 0–10, score ≤ 7 indicates cognitive impairment). The Brief Attention Test comprises digit span (3 forward trials, 2 backward trials), months of the year backward and days of the week backward (total score range 0–7, score < 5 indicates attention impairment)

*DRS-R98* Delirium Rating Scale-Revised 98 (total score range 0–46 and severity sub-score range 0–39, higher scores indicate increased likelihood and severity of delirium)

*OSLA* Observational Scale of Level of Arousal (score range 0–15, higher scores indicate more abnormal level of arousal, incorporating both reduced and increased arousal)

**Significantly different to cognitively unimpaired patients at *p* < 0.003 level after Holm–Bonferroni correction [28]

^†^Significantly different to patients with delirium at *p* < 0.003 level after Holm–Bonferroni correction [28]

### Group comparisons of response patterns in patients

In the total analysis sample of 149 patients, more patients with delirium (36/50 = 72%) than patients with dementia (21/46 = 46%; (*p* = 0.01) were unable to state that November was the month before December (Table 2). Both groups differed significantly from patients without cognitive impairment (2/53 = 4%, *p*’s < 0.001).

**Table 2:** Worse performance in MOTYB

	Delirium		Dementia		No cognitive impairment		Delirium vs dementia	Delirium vs No cognitive impairmen*t*	Dementia vs No cognitive impairmen*t*
*N* = 149	*N* = 50		*N* = 47		*N* = 53				
	*n*	%	*n*	%	*n*	%	*p*	*p*	*p*
Not meaningfully engage	23	46	5	11	0		< 0.001**	< 0.001^**^	0.02*
Not able to recite to December	26	52	7	15	0		< 0.001**	< 0.001**	0.004**
December last correct month	36	72	21	45	0		0.012*	< 0.001**	< 0.001**

Fisher exact test analyses for delirium versus dementia, delirium versus no cognitive impairment and dementia versus no cognitive impairment

*Lost significance with Holm–Bonferroni correction

**Significant with Holm–Bonferroni correction [28]

More patients in the delirium group (26/50 = 52%) than in the dementia group (7/47 = 15%) were unable to start the MOTYB by stating December as the first correct month (*p* < 0.001), and both groups differed from patients without cognitive impairment (0/53 = 0%, *p* < 0.001 and *p* < 0.004, respectively) (Table 2).

The median ‘last correct month’ was “No correct month” (IQR = November, no correct month) in the delirium group, November (IQR = September, December) in the dementia group and January (IQR = January, May) in the group without cognitive impairment. Patients with delirium stated significantly fewer last correct months than patients with dementia (*U* = − 3,802, *p* < 0.001).

More patients with delirium (23/50 = 46%) than patients with dementia (5/46 = 11%) were unable to meaningfully engage with MOTYB (*p* < 0.001; Table 2) and both groups differed from patients without cognitive impairment (0/53 = 0%; (*p* < 0.001 and *p* = 0.02, respectively).

121 patients were able to meaningfully engage with MOTYB. Of these, patients with delirium (2/27 = 7%) did not differ significantly from patients with dementia (8/41 = 20%) in ability to recite the months back to January without error (*p* = 0.29), and both groups differed from patients without cognitive impairment (35/53 = 66%; *p*’s < 0.001).

Compared to the group without cognitive impairment, patients with delirium and/or dementia more often recited the months forward instead of backward (*p* < 0.002 and *p* < 0.001), respectively, stopped part way through (*p* < 0.001 and *p* < 0.001), respectively. Stopping part way through was the most powerful discriminator between patients without cognitive impairment (none) and cognitively impaired patients, either due to delirium (18/27 = 67%, *p* < 0.001) or dementia (22/41 = 54%, *p* < 0.001). There were no significant differences between the delirium and dementia groups in frequency of any of the response patterns. None of the patients with delirium self-corrected their errors, compared to 5 (12%) in the dementia group (*p* = 0.15) and 6 (11%) in the group without cognitive impairment (*p* = 0.09), respectively (Tables 3 and 4).

**Table 3:** Reciting backwards to January for patients being able to meaningfully engage with MOTYB

	Delirium		Dementia		No cognitive impairment		Delirium vs dementia	Delirium vs no cognitive impairment	Dementia vs no cognitive impairment
*N* = 121	*N* = 27		*N* = 41		*N* = 53				
	*N*	%	*n*	%	*n*	%	*p*	*p*	*P*
December to January	2	7	8	20	35	66	0.29	< 0.001**	< 0.001**

Fisher exact test analyses for delirium versus dementia, delirium versus no cognitive impairment and dementia versus no cognitive impairment

**Significant with Holm–Bonferroni correction[28]

**Table 4:** Comparison of MOTYB response patterns in patients who are able to meaningfully engage with the task (*n* = 121)

	Delirium *N* = 27		Dementia *N* = 41		No cognitive impairment *N* = 53		Delirium vs dementia	Delirium vs no cognitive impairment	Dementia vs no cognitive impairment
	*n*	%	*n*	%	*n*	%	*p*	*p*	*P*
Error types									
Omission	7	(26)	4	(10)	6	(11)	0.10	0.12	1.00
Commission (in wrong place)	6	(22)	15	(37)	7	(13)	0.29	0.35	0.01*
Commission (non-relevant)	6	(22)	5	(12)	0	(0)	0.32	0.001**	0.01*
Repetition	5	(19)	13	(32)	11	(21)	0.27	1.00	0.24
Self-correction	0	(0)	5	(12)	6	(11)	0.15	0.09	1.00
Reciting forward	7	(26)	11	(27)	1	(2)	1.00	0.002**	< 0.001**
Stopping part way through	18	(67)	22	(54)	0	(0)	0.32	< 0.001**	< 0.001**

Fisher exact test analyses for delirium versus dementia, delirium versus no cognitive impairment and dementia versus no cognitive impairment

*Lost significance with Holm–Bonferroni correction[28]

**Significant with Holm–Bonferroni correction[28]

## Discussion

The main findings of this preliminary study are that half of the patients with delirium (46%) could not engage with MOTYB compared to only 11% of patients with dementia without delirium. This suggests that the inability to meaningfully engage with the MOTYB (i.e., not responding at all due to altered arousal or severe inattention) may be a useful indicator of possible delirium. These findings are aligned with previous reports [14, 29–31] that being so-called "untestable" (i.e., not engaging meaningfully with cognitive testing) is strongly associated with delirium, and less with dementia.

In patients able to give responses, those with delirium or dementia performed significantly worse than those without cognitive impairment. There were also differences in response patterns, in that none of the patients without cognitive impairment stopped part way through or made non-relevant commission errors. However, delirium and dementia patients able to engage with MOTYB did not generally differ in performance or response patterns such as omissions, commissions or reciting forward.

**Fig. 2:**
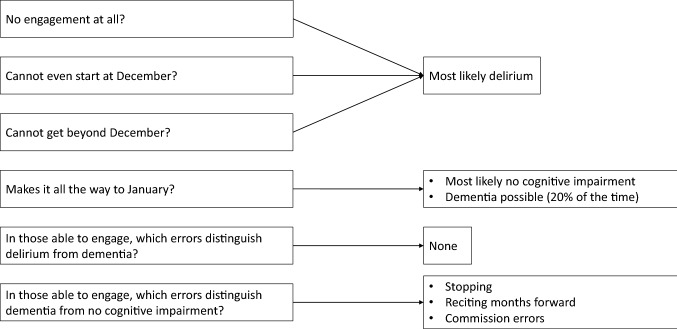
Flow chart synthesising the clinical approach from Fig 1 and Tables 2–4

Another difference between delirium and dementia patients was that none of the delirious patients self-corrected during the task. By contrast dementia patients and cognitively unimpaired patients tended to self-correct their errors. This might suggest that to self-correct mistakes requires a level of insight and self-monitoring of performance that could be lacking in people who are experiencing delirium. Additionally, patients with delirium appeared to stop earlier in the task, for example reciting months to November or October and then stop (Fig. 1). Our hypothesis about differences in scores and response patterns between dementia and delirium groups was only true for the number of recited correct months and being able to meaningfully engage with MOTYB. We did not differentiate between delirium with and without dementia in our study, and hence we cannot rule out that there might have been differences in MOTYB test performance between these two groups. Our reason for grouping delirious patients with and without dementia together was that the majority of older patients with delirium have a degree of pre-existing cognitive impairment or (often undiagnosed) dementia, but one cannot exclude with certainty undiagnosed chronic cognitive impairment in the presence of delirium (Fig. 2).

Our findings show the potential value of analysing response patterns, especially initial engagement, self-correction, and ability to continue to do the task in addition to considering exclusively the capacity to correctly recite the months until July, June or January [7]. Adapting MOTYB scoring to incorporate inability to engage in addition to a ‘correct/incorrect’ classification may add useful clinical information. Indeed, in the 4AT tool the ‘untestable’ category for the MOTYB component contributes to a possible delirium positive score [6].

This study has several limitations that should be acknowledged. The numbers of patients making individual specific errors was generally small, and some analyses were underpowered. Patients with delirium tended to stop earlier, thereby limiting the number of errors that could be produced. Participants were drawn from a case–control study in which researchers selected patients belonging to predefined clinical groups. This may overestimate the accuracy of the test performance results. We found statistically significant group differences in age despite matching on age bands, which suggests that the 10-year age bands may have been too wide. The researchers who administered MOTYB participated in the initial classification of these patients and MOTYB was used as part of the reference assessment which informed the final diagnostic grouping. This lack of blinding limits the conclusions of the present study. Due to the study design, we were unable to assess patients prior to delirium onset nor did we have pre-existing results from cognitive tests, which meant that the dementia severity of the patients with delirium and dementia was not known. Thus, we neither graded the severity of dementia nor did we differentiate between delirium with and without dementia. The findings can thus be considered preliminary and potentially informative for future more rigorous studies utilising independent, blinded assessments. Such studies could include assessment of diagnostic test accuracy (sensitivity, specificity, etc.) of the MOTYB on its own or in combination with other assessments.

This study also has several strengths. We recruited hospitalised patients representative of those who commonly undergo delirium assessments. Participant groupings were established using a reference standard with explicit operationalised diagnostic criteria incorporating neuropsychological testing, observational scales and a detailed delirium assessment instrument, the DRS-R98. With respect to the MOTYB analyses, we used an objective method of assessing response patterns based on verbatim transcripts.

These preliminary findings suggest that being unable to engage with MOTYB may have value as a possible indicator of delirium. Additionally, stopping early in the test may also be an indicator of delirium. These findings suggest that going beyond simple scoring thresholds may add to the clinical and research utility of the MOTYB. Future studies involving independent assessments, larger sample sizes, and grading of the severity of dementia patients will be informative and valuable.

## Supplementary Information

Below is the link to the electronic supplementary material.

Supplementary file1 (PPTX 39 KB)

Supplementary file2 (DOCX 15 KB)

## Data Availability

On request.

## Abbreviations

AMJM: Alasdair MacLullich
CC: Caoimhe Clarke
DS: David Stott
EN: Eva Nouzova
LS: Lisa-Marie Süßenbach
ND: Nikki Duncan
ZT: Zoë Tieges
AMT: Abbreviated Mental Test
DRS-R98: Delirium Rating Scale—Revised 98
MOTYB: Months of the Year Backward
OMCT: Short Orientation, Memory and Concentration Test
OSLA: Observational Scale of Level of Arousal
RASS: Richmond Agitation–Sedation Scale
