# Supplementary material for: Comparing performance on the Months of the Year Backwards test in hospitalised patients with delirium, dementia, and no cognitive impairment: an exploratory study
Source: Eur Geriatr Med. 2021 Jun 22;12(6):1257–65. doi: 10.1007/s41999-021-00521-4 (PMC8626373; doi:10.1007/s41999-021-00521-4)
Supplement: Supplementary file 1 — Supplementary file1 (PPTX 39 KB) [file 41999_2021_521_MOESM1_ESM.pptx]

## Slide 1
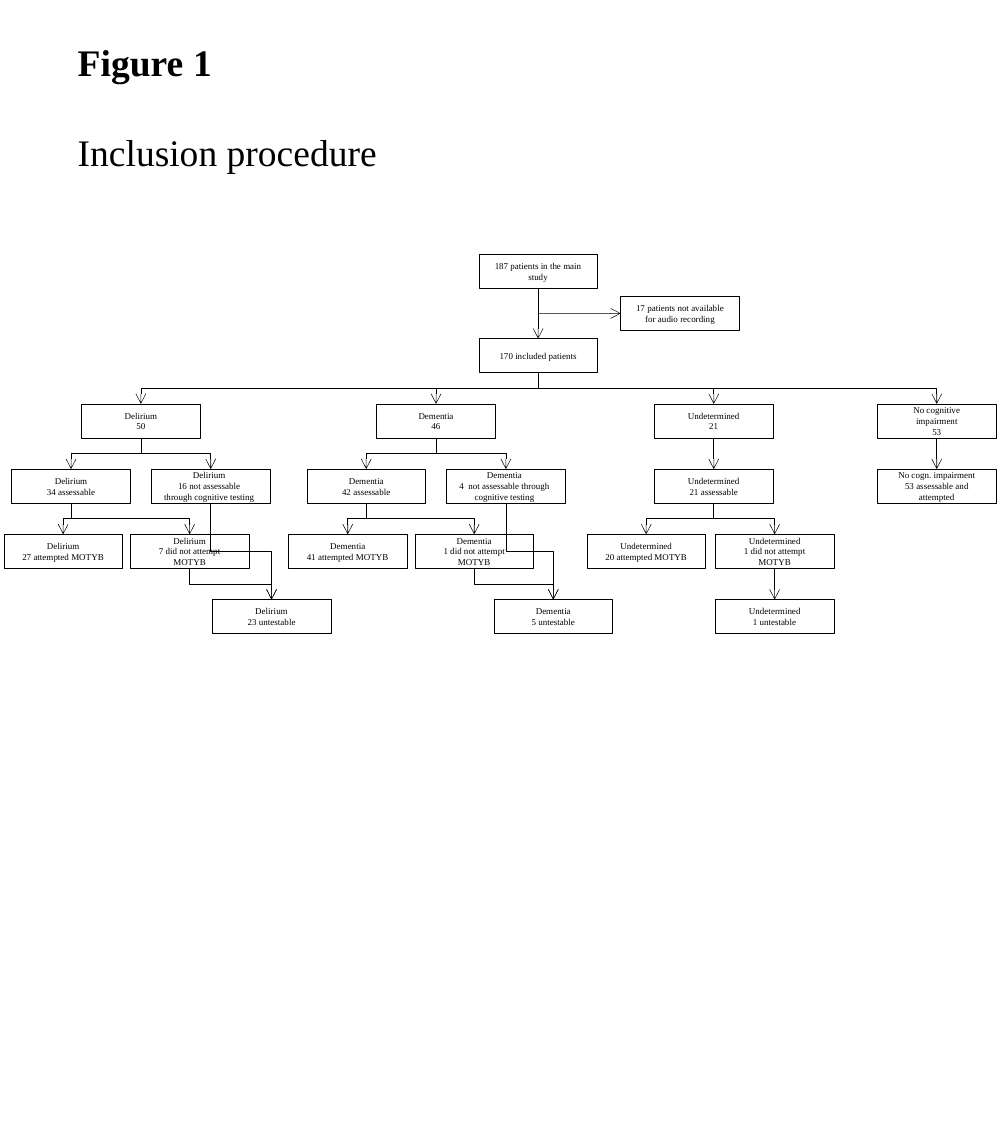

Figure 1
Inclusion procedure
187 patients in the main study
17 patients not available for audio recording
170 included patients
Delirium
50
Dementia
46
Undetermined
21
No cognitive impairment
53
Delirium
34 assessable
Delirium
16 not assessable through cognitive testing
Dementia
42 assessable
Dementia
4 not assessable through cognitive testing
Undetermined
21 assessable
No cogn. impairment
53 assessable and attempted
Delirium
27 attempted MOTYB
Delirium
7 did not attempt MOTYB
Dementia
41 attempted MOTYB
Dementia
1 did not attempt MOTYB
Undetermined
20 attempted MOTYB
Undetermined
1 did not attempt MOTYB
Delirium
23 untestable
Dementia
5 untestable
Undetermined
1 untestable
