# Supplementary material for: Comparing performance on the Months of the Year Backwards test in hospitalised patients with delirium, dementia, and no cognitive impairment: an exploratory study
Source: Eur Geriatr Med. 2021 Jun 22;12(6):1257–65. doi: 10.1007/s41999-021-00521-4 (PMC8626373; doi:10.1007/s41999-021-00521-4)
Supplement: Supplementary file 2 — Supplementary file2 (DOCX 15 KB) [file 41999_2021_521_MOESM2_ESM.docx]

## **Online Table 1**

*Pattern Characteristics*

| Pattern | Characteristics |
| --- | --- |
| Omission | Skipping a month (e.g. December, November, September, August, July, June, May, April, March, February, January) |
| Commission in the wrong place | Saying a month in the wrong place (e.g. December, November, October, September, July, August, June, May, April, March, February, January) |
| Non-relevant commission | Saying a non-relevant word during the test (e.g. December, November, October, 19, 18, 17, September, August, July, June, May, April, March, February, January) |
| Repetition | Saying a month more than once (e.g. December, November, October September, August, July, July, June, May, April, March, February, January) |
| Self-correction | Realising an error was made and correcting this immediately (e.g. December, November, September, sorry no October then September, August, July, June, May, April, March, February, January) |
| Reciting the months forward | Changing reciting direction (E.g. December, November, January, February, March, April, May, June, July, August, September, October, November, December) |
| Stopping part way through the task | Not finishing reciting (e.g. December, November, October, Sorry I can’t do the rest - or falling asleep) |
| Not being able to meaningfully engage with the task | Not responding at all or answers with non-relevant topics |
